# Anthracycline Treatments and the Presence of Tumor Cells Synergistically Modify the Composition of Macrophage Subpopulations in the Co-Culture System

**DOI:** 10.3390/ijms26189202

**Published:** 2025-09-20

**Authors:** Viktória Jenei, Zsuzsa Muszka, Ádám Stigelmayer, Zsuzsanna Debreceni, Attila Bácsi, Anett Mázló, Gábor Koncz

**Affiliations:** 1 Department of Immunology, Faculty of Medicine, University of Debrecen, Egyetem Square 1, 4032 Debrecen, Hungary; jenei.viki97@gmail.com (V.J.);; 2 Gyula Petrányi Doctoral School of Allergy and Clinical Immunology, University of Debrecen, Egyetem Square 1, 4032 Debrecen, Hungary; 3 Doctoral School of Molecular Cell and Immune Biology, University of Debrecen, Egyetem Square 1, 4032 Debrecen, Hungary

**Keywords:** monocyte, macrophage, cancer, immunomodulation, doxorubicin, epirubicin

## Abstract

In addition to killing malignant cells, effective cancer therapies must also promote the development of an immunostimulatory tumor microenvironment (TME). Macrophages are the most abundant immune cell population within the TME. These highly plastic cells play key roles in tumor progression, chronic inflammation, immunosuppression, and metastasis. Although increasing research efforts focus on manipulating macrophage functions, relatively little is known about how standard anticancer strategies, especially chemotherapeutic agents, influence the composition, polarization state, and functional behavior of macrophage subpopulations. Chemotherapeutic agents remain a primary treatment option for many types of cancer, including breast and cervical cancers. In this study, we used epirubicin and doxorubicin at near-therapeutic concentrations and examined their effects on macrophage functions in co-culture with MDA-MB-231 breast cancer and HeLa cervical cancer cell lines. We demonstrated that the presence of tumor cells led to increased expression of the M2 macrophage marker CD206, a change that was reduced by both chemotherapeutic agents. The production of macrophage-derived chemokines, such as IP-10 and IL-8, was also altered by tumor presence and drug exposure. A striking finding was that the co-presence of chemotherapeutic agents and MDA-MB-231 cells synergistically altered macrophage motility. This effect was not observed in monocultures. Furthermore, the presence of tumor cells reduced the susceptibility of pro-inflammatory M1 macrophages to drug-induced cell death. These results indicate that chemotherapy can reshape the macrophage landscape in the TME. We highlight that the combined effects of tumor cell presence and chemotherapy modulate the composition, phenotype, and migration of macrophage subtypes differently than either factor alone.

## 1. Introduction

The tumor microenvironment (TME) is a key regulator of tumor growth, progression, and metastasis. Therefore, optimal therapeutic strategies should aim not only to directly target tumor cells, but also to enhance the immunogenicity of the tumor microenvironment. Among the innate immune cells recruited to the tumor site, macrophages represent the most abundant cell population within the microenvironment of solid tumors [1]. Macrophages are involved in all stages of tumor progression. They help maintain chronic inflammation and contribute to immunosuppression. They also induce angiogenesis and support epithelial–mesenchymal transition and are also known to play a role in metastasis [2]. These effects are further modulated by the hypoxic nature of the TME, which profoundly influences macrophage behavior by stabilizing hypoxia-inducible factors (HIFs) and promotes their differentiation into an immunosuppressive phenotype [3].

Tumor macrophages (TAMs) exist along a dynamic spectrum between pro-inflammatory M1 and anti-inflammatory M2 polarization states, with potential for repolarization. While M2-like TAMs are predominantly associated with pro-tumor functions, M1-like TAMs are typically considered anti-tumor cells, although under certain conditions their pro-inflammatory effects may also contribute to tumorigenesis [4]. As highly plastic cells, macrophages undergo continuous functional reprogramming in response to signals within the tumor environment. Modulating macrophage subpopulations within the tumor microenvironment has been a long-standing therapeutic objective. Strategies such as regulating the differentiation of macrophage subpopulations, directing the migration of monocytes and/or macrophage subtypes, and selective killing of M1- or M2-like macrophages are all considered therapeutic options [5]. However, relatively little is known about how current therapies, such as chemotherapy, affect these macrophage properties.

Chemotherapy remains a cornerstone treatment for various malignancies, including cervical and breast cancers, and is often administered as a first-line therapy. In addition, chemotherapy treatments become important in combination with other therapeutic treatments, enhancing the effectiveness of biological and immunotherapies [6]. To optimize such combination strategies, it is essential to understand how chemotherapy influences the TME, particularly the macrophages residing within it [7]. Chemotherapy acts not only on tumor cells but also on surrounding immune cells, thereby reshaping their interactions. Chemotherapeutic agents can modulate macrophages through three main mechanisms: (1) altering macrophages phenotype; (2) inducing the recruitment of monocytes or macrophages to the tumor site; (3) modifying the survival capacity of specific macrophage subpopulations.

In our previous work, we examined and described the effects of two anthracyclines—epirubicin and doxorubicin—on macrophage monocultures [8]. In the current project, we investigated the complex effects of these chemotherapeutic agents on macrophage subpopulations in in vitro macrophage–tumor cell co-cultures. Specifically, we analyzed how epirubicin and doxorubicin—frequently used in the first-line treatment of cervical and breast cancer—affect macrophages cultured with triple-negative MDA-MB-231 breast cancer cells and HeLa cervical cancer cells. We demonstrated how tumor cells influence macrophage differentiation/polarization, chemotaxis, and survival, and conversely, how the presence of macrophages affects tumor cell sensitivity to chemotherapy.

## 2. Results

### 2.1. The Presence of Tumor Cells Increases CD206 Expression on Macrophages

**Figure 1:**
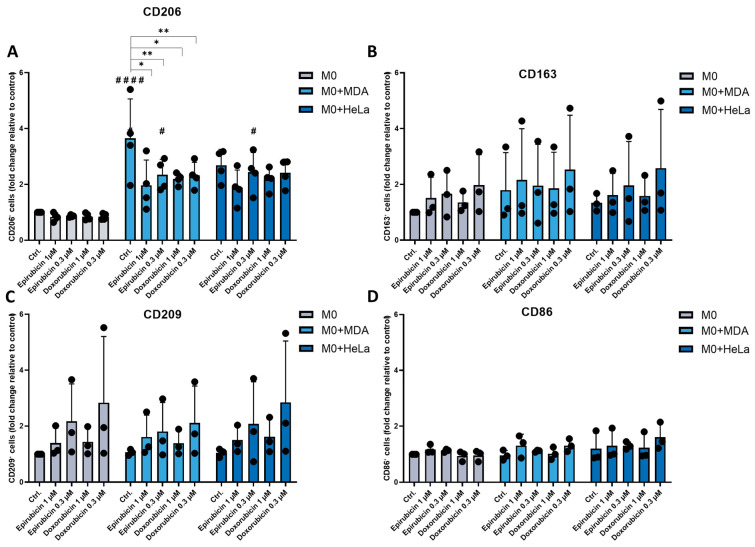
Co-culturing macrophages with tumor cells and treating them with chemotherapeutic agents modifies the pattern of cell surface marker expression. CD14^+^ monocytes were cultured with 50 ng/mL recombinant M-CSF for 5 days to generate M0 macrophages. These macrophages were labeled with 10 ng/mL CellTracker™ Green CMFDA dye and were co-cultured with unlabeled MDA-MB-231 or HeLa cell lines at a 1:1 ratio for 24 h. Following this incubation, cells were treated with chemotherapeutic agents (either doxorubicin or epirubicin) for 24 h. Cell surface expression of (**A**) CD206, (**B**) CD163, (**C**) CD209, and (**D**) CD86 on macrophages was analyzed by flow cytometer. Mean values of median fluorescence intensity and the percentage of cells positive for the measured surface markers were calculated relative to the control from at least three independent experiments and are presented as mean ± SD. Statistical analysis was performed using ANOVA followed by Bonferroni’s post hoc test for comparisons. Results are expressed as mean ± standard deviation. Differences were considered statistically significant at *p* < 0.05. Statistical differences are indicated by # *p* < 0.05 and #### *p* < 0.0001 versus untreated control cells, and * *p* < 0.05 and ** *p* < 0.01 versus treated counterparts.

To investigate how chemotherapeutic agents affect macrophage differentiation in the presence of tumor cells, we co-cultured unpolarized macrophages (M0) with triple negative MDA-MB-231 breast cancer cells or HeLa cervical cancer cells and analyzed the expression of macrophage surface markers following treatment with doxorubicin or epirubicin. To minimize cytotoxicity to M0 macrophages, we applied approximately one-tenth of the maximum plasma concentrations of the highest clinically recommended doses [9]. We assessed the expression of surface markers characteristic of both pro-inflammatory (CD86) and anti-inflammatory (CD163, CD206, CD209) phenotypes on macrophages cultured either in monocultures or in co-culture with tumor cells. Expression of CD163, CD209 and CD86 markers was not affected by the presence of tumor cells or by chemotherapeutic treatments, doxorubicin or epirubicin. In contrast, CD206 expression was upregulated on macrophages co-cultured with either tumor cell line. However, treatment with either epirubicin or doxorubicin reduced CD206 expression in macrophages co-cultured with MDA-MB-231 cells (Figure 1).

By examining the RNA expression of genes characteristic of M1 and M2 macrophages, we demonstrated that in the coculture of macrophages and tumor cells, but not in any of the monocultures, the expression levels of both M1 markers (CD80, CD86) and M2 markers (*Arg1*) increased following chemotherapy treatments. Highlighting the unique properties of different tumor cells, *PPARγ* levels were increased after treatments only in cocultures of Hela cells and macrophages, but not in the case of coculture of macrophages with MDA cells (Supplementary Figure S1).

### 2.2. The Presence of Tumor Cells or Chemotherapeutic Agents Regulates the Production of IL-8 and IP-10 by Macrophages

**Figure 2:**
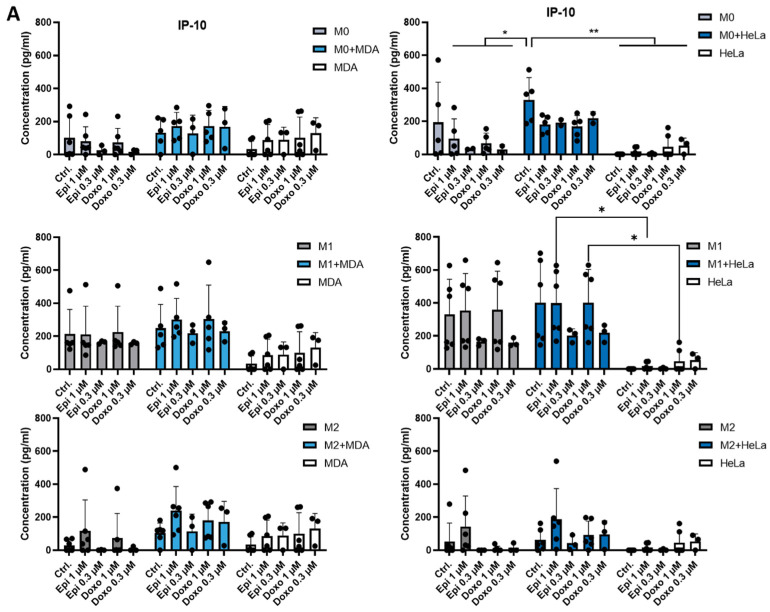

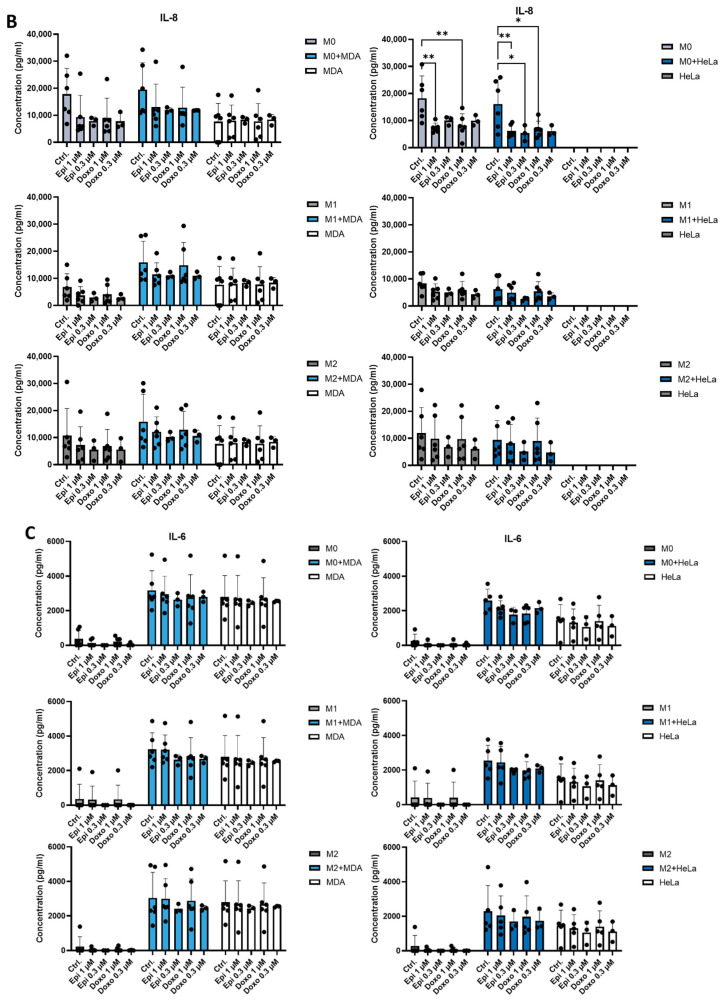
Co-culture of macrophage subpopulations with tumor cells and treatment with chemotherapeutic agents modulate cytokine production by macrophages. CD14^+^ monocytes were cultured with 50 ng/mL recombinant M-CSF for five days to generate M0 macrophages. These macrophages were further polarized into M1 or M2 phenotypes using LPS (50 ng/mL) and IFNγ (20 ng/mL) or IL-4 (20 ng/mL), IL-10 (20 ng/mL) and TGF-β (20 ng/mL), respectively. In vitro-differentiated human M0, M1, and M2 macrophages were co-cultured with MDA-MB-231 or HeLa tumor cells at a 1:1 ratio for 24 h. Co-cultures were then treated with doxorubicin or epirubicin. After an additional 24 h, concentrations of (**A**) IP-10, (**B**) IL-8 and (**C**) IL-6 secreted by macrophages were measured by ELISA. Statistical analysis was performed using ANOVA followed by Bonferroni’s post hoc test. Results are presented as mean ± standard deviation (SD). Differences were considered statistically significant at *p* < 0.05. Statistical differences are indicated as * *p* < 0.05 and ** *p* < 0.01 versus treated counterparts.

Next, we investigated how tumor cells influence macrophage functionality, focusing on M0, M1 and M2 macrophage subsets. M0 cells were differentiated for 5 days in the presence of M-CSF and subsequently polarized for 24 h with LPS and IFNγ to generate M1 macrophages, or with IL-4, IL-10, and TGF-β to generate M2 macrophages. Cytokine production was assessed in monocultures of M0, M1, and M2 macrophages, as well as in co-cultures with MDA-MB-231 or HeLa cancer cell lines. We also tested the effects of doxorubicin and epirubicin on cytokine production. The presence of tumor cells modified macrophage function: a significant difference in the production of IP-10 was observed between anthracycline-treated M0 monocultures and M0-HeLa co-cultures (Figure 2A). However, these differences were not observed in M1 or M2 macrophages. In some conditions, chemotherapeutic agents also modulated chemokine production by reducing IL-8 levels in M0 macrophage monocultures and M0-HeLa co-cultures (Figure 2B). However, under other conditions we could not detect any chemotherapy-related change. Consistent with previous findings [10,11], both MDA-MB-231 and HeLa cells produced high amounts of IL-6, which remained unchanged in the presence of macrophages or after chemotherapy treatments (Figure 2C).

### 2.3. Anthracyclines Enhance the Migratory Potential of Macrophage Subpopulations Towards Tumor Co-Cultures

**Figure 3:**
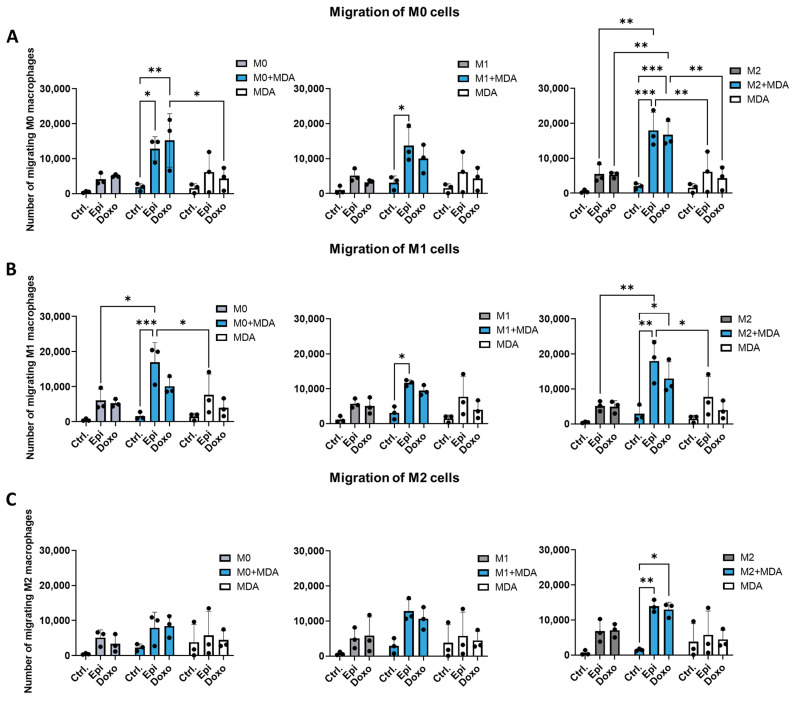
Co-culture with tumor cells and chemotherapy treatment influences the migratory potential of macrophage subpopulations. CD14^+^ monocytes were cultured with 50 ng/mL recombinant M-CSF for five days to generate M0 macrophages. These were subsequently polarized into M1 and M2 phenotypes using LPS (50 ng/mL) and IFNγ (20 ng/mL), or IL-4 (20 ng/mL), IL-10 (20 ng/mL), and TGF-β (20 ng/mL), respectively. In vitro-differentiated human M0, M1, and M2 macrophages were co-cultured with MDA-MB-231 tumor cells at a 1:1 ratio in 0.2 mL medium in 96-well tissue culture plates for 24 h. Co-cultures were then treated with chemotherapeutic agents (3 μM doxorubicin or 3 μM epirubicin) for an additional 24 h. (**A**) M0, (**B**) M1, and (**C**) M2 macrophages were labeled with 10 ng/mL CellTracker™ Green CMFDA dye prior to migration assays Migration was assessed by quantifying cells that crossed a semipermeable membrane insert over 30 min. Statistical analysis was performed using ANOVA followed by Bonferroni’s post hoc test. Results are presented as mean ± standard deviation (SD). Differences were considered statistically significant at *p* < 0.05. Statistical differences are indicated as * *p* < 0.05, ** *p* < 0.01 and *** *p* < 0.001 versus treated counterparts.

Controlling the cellular composition of the TME by influencing macrophage migration is a long-standing therapeutic objective [12]. However, the effect of chemotherapy on macrophage migration remains poorly understood. Chemotherapeutic agents may regulate the migratory potential of macrophage subtypes either directly or indirectly by altering tumor cell–macrophage interactions. To investigate this, we assessed the chemotaxis of chemotherapy-treated M0, M1, and M2 macrophages toward: tumor cell monocultures (MDA-MB-231 or HeLa), and tumor–macrophage co-cultures. Since all three macrophage types may be present within the TME, we established co-cultures of MDA-MB-231 or HeLa tumor cells with M0, M1, or M2 macrophages. In the absence of chemotherapeutic treatment, macrophage migration toward tumor co-cultures did not differ significantly from that observed with monocultures. Similarly, epirubicin or doxorubicin treatment did not alter the migratory behavior of M0, M1, or M2 macrophages in monocultures of either macrophages or tumor cells (Figure 3). In contrast, anthracycline treatment of MDA-MB-231–macrophage co-cultures consistently increased the migration of all three macrophage subsets, resulting in statistically significant changes in most conditions (Figure 3). After chemotherapy treatment, migration of M0 and M1 cells increased towards co-cultures of MDA-MB-231 cells with any macrophage subtype. (Figure 3A,B). Interestingly, M2 macrophages exhibited significantly increased migration only toward co-cultures containing M2 macrophages, even after treatment with chemotherapeutic agents (Figure 3C). No significant differences were observed between doxorubicin and epirubicin regarding their effects on macrophage migration. Although similar trends were seen with HeLa cell co-cultures, neither HeLa monocultures nor HeLa–macrophage co-cultures influenced significantly macrophage migration under any tested conditions (Supplementary Figure S2). These findings suggest that chemotherapeutic modulation of macrophage migration is context-dependent and occurs specifically in co-culture systems involving MDA-MB-231 tumor cells, but not in monocultures.

### 2.4. Co-Culture with MDA-MB-231 Cells Reduces the Sensitivity of M1 Macrophages to Anthracycline Treatment

**Figure 4:**
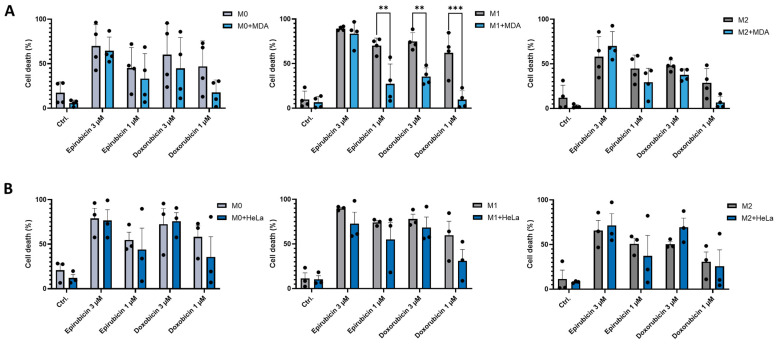
The presence of tumor cells influences the chemosensitivity of macrophages. CD14^+^ monocytes were cultured with 50 ng/mL recombinant M-CSF for 5 days to generate M0 macrophages. In vitro-differentiated human M0, M1, and M2 macrophages were loaded with 10 ng/mL CellTracker™ Green CMFDA dye and incubated for 30 min. After extensive washing, macrophages were co-cultured with unlabelled (**A**) MDA-MB-231 or (**B**) HeLa tumor cell lines at a 1:1 ratio for 24 h. Co-cultures were then treated with chemotherapeutic agents (doxorubicin or epirubicin) for an additional 24 h. Macrophage cell death was assessed by propidium iodide (PI) staining. The percentage of macrophage cell death was calculated from at least three independent experiments and is presented as mean ± standard deviation (SD). Statistical analysis was performed using ANOVA followed by Bonferroni’s post hoc test for comparisons. Differences were considered statistically significant at *p* < 0.05. Statistical significance is indicated as ** *p* < 0.01 and *** *p* < 0.001 compared to treated counterparts.

Increasing evidence suggests that chemotherapy modulates the immune microenvironment. However, the effects of chemotherapeutic agents on the survival of distinct macrophage subpopulations remain poorly characterized. Since interactions between macrophages and tumor cells can influence the chemosensitivity of both cell types, we examined the sensitivity of each cell type when cultured together. In monocultures, all macrophage subpopulations (M0, M1, and M2) were susceptible to both epirubicin and doxorubicin. In contrast, a significant reduction in chemosensitivity was observed for M1 macrophages in co-culture with MDA-MB-231 cells compared to monocultures. This decrease in cell death sensitivity after epirubicin treatment was observed only when a suboptimal dose was used (Figure 4A). No significant differences in chemosensitivity of the treated macrophage subpopulations were observed in co-cultures with HeLa cells (Figure 4B).

**Figure 5:**
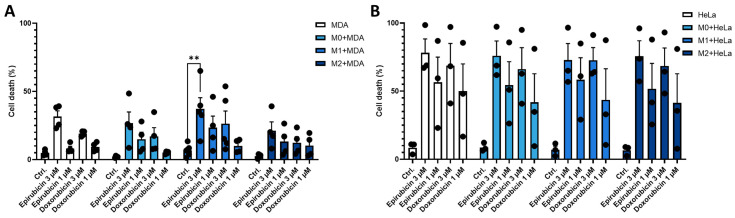
Effect of macrophages on the chemosensitivity of tumor cell lines. CD14^+^ monocytes were cultured with 50 ng/mL recombinant M-CSF for 5 days to generate M0 macrophages. In vitro-differentiated human M0, M1, and M2 macrophages were loaded with 10 ng/mL CellTracker™ Green CMFDA dye and incubated for 30 min. After extensive washing, macrophages were co-cultured with unlabeled (**A**) MDA-MB-231 or (**B**) HeLa tumor cell lines at a 1:1 ratio for 24 h. Co-cultures were then treated with the chemotherapeutic drugs (doxorubicin or epirubicin) for an additional 24 h. Tumor cell death was assessed by propidium iodide (PI) staining. There was no significant difference between the corresponding sample pairs in the absence of macrophages. The percentage of tumor cell death was calculated from at least three independent experiments and is presented as mean ± standard deviation (SD). Statistical analysis was performed using ANOVA followed by Bonferroni’s post hoc test. Differences were considered statistically significant at *p* < 0.05. Statistical significance is indicated as ** *p* < 0.01 compared to treated counterparts.

In co-culture systems, we examined whether the presence of different macrophage subpopulations affects the chemosensitivity of tumor cells. No differences in chemotherapeutic agent–induced cell death were observed in either tumor cell line, whether cultured alone or in co-culture with macrophages (Figure 5).

## 3. Discussion

As highlighted by the success of immunotherapies, anticancer strategies should target components of the TME in addition to tumor cells to promote optimal antitumor immunity. Macrophages have emerged as one of the most promising targets for therapeutic modulation of tumor immunity due to their high abundance in the TME, functional plasticity, and responsiveness to pharmacological intervention [5,13]. Several therapeutic approaches are currently exploring the regulation of macrophage functions, by targeting differentiation [14], chemotaxis [15], or selective survival of specific subtypes [16]. However, how currently used therapies—particularly chemotherapy—affect macrophages within the TME remains largely unexplored. Although some studies have investigated the influence of tumor cells or chemotherapeutic agents on the differentiation of monocytes into macrophages [8,17] less is known about how therapeutic agents affect macrophages already embedded in the tumor environment.

To address this, we examined the effects of chemotherapy in co-culture systems to better understand how chemotherapeutic agents regulate the dynamic crosstalk between tumor cells and macrophages. We focused on anthracyclines—doxorubicin and epirubicin—which are widely used as first-line chemotherapeutic agents in breast cancer treatment. Although they are not considered standard first-line drugs for cervical cancer, both have been clinically applied in selected cases of recurrent or metastatic cervical cancer [18]. Given their well-established immunomodulatory effects, we investigated their impact on macrophage behavior in co-cultures involving triple-negative MDA-MB-231 breast cancer cells and HeLa cervical cancer cells, to model tumor–macrophage interactions within the TME. Accordingly, we first investigated how the differentiation of M0 macrophages is altered by chemotherapeutic agents in the presence of tumor cells. Both tumor cell lines increased the expression of CD206, a key anti-inflammatory marker associated with M2-like macrophages. This increase was attenuated by doxorubicin and epirubicin treatment in co-cultures with MDA-MB-231 cells. However, the expression of other M1 (CD86) and M2 markers (CD209, CD163) remain unchanged.

CD206, a mannose receptor, involved in phagocytosis, immunosuppression, tissue repair, and has also been implicated in promoting metastasis and angiogenesis within the tumor microenvironment [19]. Our results therefore suggest that tumor cells influence macrophage differentiation in a manner that does not conform to the classical M1/M2 polarization paradigm, but results in modifications of specific functions of tumor-associated macrophages.

Functionally, our results showed that IL-6 production, a cytokine critical for tumor immunity, remained unchanged under the co-culture conditions. However, the levels of IP-10 and IL-8 produced by macrophages was modestly affected by the presence of tumor cells and chemotherapeutic treatment. Our results are consistent with previous studies that reported only modest changes in cytokine production in macrophages co-cultured with tumor cells [8,17,20].

The most intense changes were observed in macrophage migration. While tumor cells or chemotherapy alone had minimal impact on macrophage mobility, their combination—particularly with MDA-MB-231 cells—significantly enhanced macrophage movement towards the co-cultured cells. Our findings indicated that all the macrophage subtypes interacting with tumor cells contributed critically to the overall migratory response.

Active recruitment of specific macrophage subpopulations and the subsequent shift in cellular composition are crucial in tumor metastasis [21]. Consequently, regulating the migration of macrophage subpopulations into and out of the TME remains a long-term therapeutic goal. Similarly, chemotherapy-induced metastases—previously an overlooked phenomenon—has emerged as a novel concept in cancer treatment [22]. Chemotherapy’s influence on metastasis may partly stem from its effects on macrophages, as it has been shown to increase perivascular macrophage density—thereby promoting tumor revascularization and recurrence in both murine and human models [12,23]. Our results suggests that macrophages may respond to chemoattractants secreted by other macrophages sensing drug-treated tumor cells; however, further investigations are needed to identify these factors. These findings highlight the importance of studying the effects of tumor cells and chemotherapy on macrophages together, which may be essential for designing effective monotherapy and combination treatment strategies.

Since macrophage sensitivity to anthracycline-induced cell death falls within a similar dose range as that of MDA-MB-231 and HeLa cell lines, we examined both cell types to determine whether chemotherapeutic agents in co-culture settings altered tumor sensitivity or the distribution of macrophage subtypes. In our study, the presence of macrophages had no impact on the sensitivity of tumor cells to anthracycline treatment. In contrast, M1 macrophages exhibited reduced susceptibility to cell death when co-cultured with MDA-MB-231 cells. However, this effect was not observed in co-cultures with HeLa cells.

The ratio of macrophage subpopulations in the TME can be a critical determinant for immune regulation. Selective elimination of macrophage subtypes represents a potential target for therapeutic intervention [16,24]. Our results are novel in this area, demonstrating that currently used therapies can also modify the composition of the TME. In addition, our findings suggest that certain tumor types may secrete survival-promoting factors that preferentially support M1 macrophages, highlighting novel avenues for further investigation.

Targeting macrophages—as monotherapy or in combination with immunotherapies—represent a promising strategy for enhancing treatment outcomes. Collectively, our findings demonstrate that chemotherapeutic agents reshape the composition of macrophage subpopulations within the TME by altering their phenotype, recruitment and chemoresistance. These changes may, in turn, influence tumor progression, metastasis, and both acute and long-term responses to anthracycline treatment. Moreover, our results emphasize the need to consider macrophage polarization when optimizing drug dosages and designing combination therapies.

## 4. Materials and Methods

### 4.1. Differentiation of Human Macrophages

Heparinized, leukocyte-enriched buffy coats were obtained from healthy blood donors drawn at the Regional Blood Center of the Hungarian National Blood Transfusion Service (Debrecen, Hungary) in accordance with the written approval from the Director of the National Blood Transfusion Service and the Regional and Institutional Research Ethical Committee of the University of Debrecen, Faculty of Medicine (Debrecen, Hungary). Written, informed consent was obtained from all donors prior to blood collection, and donor data were processed and stored in compliance with European Union data protection regulations. Peripheral blood mononuclear cells (PBMCs) were isolated from buffy coats by density gradient centrifugation using Ficoll-Paque Plus (Amersham Biosciences, Amersham, UK). Monocytes were subsequently purified from PBMCs by positive selection using immunomagnetic cell separation with anti-CD14-conjugated microbeads (Miltenyi Biotec, Bergisch Gladbach, Germany), according to the manufacturer’s instructions. Following separation on a VarioMACS magnet, 96–99% of the cells were confirmed to be CD14^+^ monocytes by flow cytometry. Isolated monocytes were seeded at a concentration of 0.5 × 10^6^ cell/mL in RPMI-1640 (Sigma-Aldrich, St. Louis, MO, USA; #R5886). To induce macrophage differentiation, 50 ng/mL M-CSF (PeproTech, Cranbury, NJ, USA) was added to the cells on the day of separation. On day 2, half of the medium was replaced with fresh RPMI-1640 containing 50 ng/mL M-CSF.

M1 and M2 macrophages were generated as we previously described [8]. Briefly, monocytes differentiated in the presence of M-CSF were stimulated on day 5 of differentiation for 24 h with either lipopolysaccharide (50 ng/mL ultrapure LPS, InvivoGen, San Diego, CA, USA) and IFNγ (20 ng/mL, PeproTech, Cranbury, NJ, USA) to induce M1 polarization or IL-4 (20 ng/mL, PeproTech), IL-10 (20 ng/mL, PeproTech) and TGF-β (20 ng/mL, PeproTech) to induce M2 phenotype.

### 4.2. Cell Lines

MDA-MB-231 (human breast adenocarcinoma) and HeLa (human cervical cancer) cell lines were cultured in RPMI-1640 (Sigma-Aldrich, St. Louis, MO, USA; R5886) at 37 °C in a humidified atmosphere containing 5% CO_2_. All media were supplemented with 10 % fetal bovine serum (FBS), 292 mg/L l-glutamine, and 1% antibiotic-antimycotic solution.

### 4.3. Direct Co-Culture

Co-cultures were established by combining HeLa or MDA-MB-231 tumor cell lines with macrophage subpopulations (M0/M1/M2). To distinguish macrophages from tumor cells, immune cells were loaded with CellTracker™ Green CMFDA dye (Thermo Fischer Scientific, Waltham, MA, USA) at 37 °C for 30 min (Supplementary Figure S3). After extensive washing, the labeled macrophages were co-cultured with unlabeled tumor cells at a 1:1 ratio in 0.5 mL medium in 24-well tissue culture plates for 24 h or in 0.2 mL medium in 96-well plates for migration assays. After this incubation period, chemotherapeutic agents—doxorubicin (0.3 μM, 1 μM, 3 μM Teva) or epirubicin (0.3 μM, 1 μM, 3 μM, Teva)—were added to the cell cultures and incubated for an additional 24 h.

### 4.4. Detection of Cell Surface Markers

Phenotyping of CellTracker-positive macrophages in tumor cell co-cultures treated with chemotherapeutic agents was performed using flow cytometry, following surface staining with CD209/DC-SIGN-phycoerythrin (PE), CD86-PE, CD163-PE, CD206-allophycocyanin (APC) (all antibodies from BioLegend) (Supplementary Figure S4). Fluorescence intensities were measured using a NovoCyte 2000R Flow Cytometer (Agilent/Acea Biosciences Inc., San Diego, CA, USA), and data were analyzed with FlowJo software version X.0.7 (Tree Star, Ashland, OR, USA).

### 4.5. Quantitative Real-Time PCR

Macrophage–tumor cell co-cultures were established as described above, where M0 macrophages were co-cultured with tumor cells and subsequently treated with chemotherapeutic agents. Total RNA was isolated from 2 × 10^6^ cells using Tri reagent (Molecular Research Center, Inc., Cincinnati, OH, USA). One microgram of total RNA was treated with DNase I (Thermo Fisher Scientific, Waltham, MA, USA) then reverse transcribed into cDNA using the High-Capacity cDNA RT Kit of Applied Biosystems (Foster City, CA, USA). Gene expression assays were purchased from Integrated DNA Technologies (Coralville, IA, USA) for *CD80, CD86, Arg1, and PPARγ* (Supplementary Figure S1). Quantitative PCR was performed using the ABI StepOne Real-Time PCR System (Applied Biosystems) and cycle threshold values were determined using the StepOne v2.1 Software (Applied Biosystems). The relative amount of mRNA was obtained by normalizing to the H36B4 (Integrated DNA Technologies, Coralville, IA, USA) housekeeping gene in each experiment.

### 4.6. ELISA

Co-cultures were established as described above; control cells received medium alone. Twenty-four hours after chemotherapy treatment, culture supernatants were collected and the concentrations of cytokine IL-6, and the chemokines IP-10 and IL-8 were measured by ELISA using OptEIA kits (BD Biosciences, Franklin Lakes, NJ, USA), according to the manufacturer’s instructions.

### 4.7. Boyden-Chamber Migration Assay

For chemotaxis measurements, in the Boyden chamber, 5 × 10^4^ CellTracker Green-labeled macrophages were added to the upper chamber. The lower chamber contained tumor co-cultures pre-treated with chemotherapeutic agents as described above. After 1 h of incubation at 37 °C, cells that had migrated into the lower chamber were collected and quantified on a NovoCyte 2000R flow cytometer (Agilent/Acea Biosciences Inc.); data were analyzed with FlowJo software version X.0.7 (Tree Star).

### 4.8. Determination of Cell Viability

Co-cultures were established as described above. Total cell death in co-cultures was quantified by propidium iodide (PI, Sigma-Aldrich) uptake, reflecting loss of membrane integrity. Immediately before flow-cytometric analysis, cells were stained with PI (10 μg/mL). Samples were acquired on a NovoCyte 2000R flow cytometer (Agilent/Acea Biosciences Inc.) and analyzed with FlowJo software (Tree Star). All experiments were performed in at least three independent replicates, and cell viability is reported as the percentage of live cells relative to untreated controls cells.

### 4.9. Statistical Analysis

Statistical analyses were performed using Excel (Microsoft Corporation, Redmond, WA, USA) and GraphPad Prism Version 6.0 (GraphPad Software Inc., La Jolla, CA, USA). Data are presented as mean ± standard deviation (SD). Differences were considered statistically significant at *p* < 0.05. In the statistical analysis, ANOVA followed by Bonferroni’s post hoc test was used for the comparisons. Significance was indicated as ^#^ *p* < 0.05, ^##^ *p* < 0.01 and ^###^ *p* < 0.001 compared to untreated control cells and as * *p* < 0.05, ** *p* < 0.01 and *** *p* < 0.001 compared to treated counterparts.

## Data Availability

Data are available from the corresponding authors upon reasonable request.

## Supplementary Materials

The following supporting information can be downloaded at: https://www.mdpi.com/article/10.3390/ijms26189202/s1.
