# Supplementary material for: Anthracycline Treatments and the Presence of Tumor Cells Synergistically Modify the Composition of Macrophage Subpopulations in the Co-Culture System
Source: Int J Mol Sci. 2025 Sep 20;26(18):9202. doi: 10.3390/ijms26189202 (PMC12470912; doi:10.3390/ijms26189202)
Supplement: Supplementary file 1 [file ijms-26-09202-s001.zip › ijms-3752828-supplementary.pptx]

## Slide 1
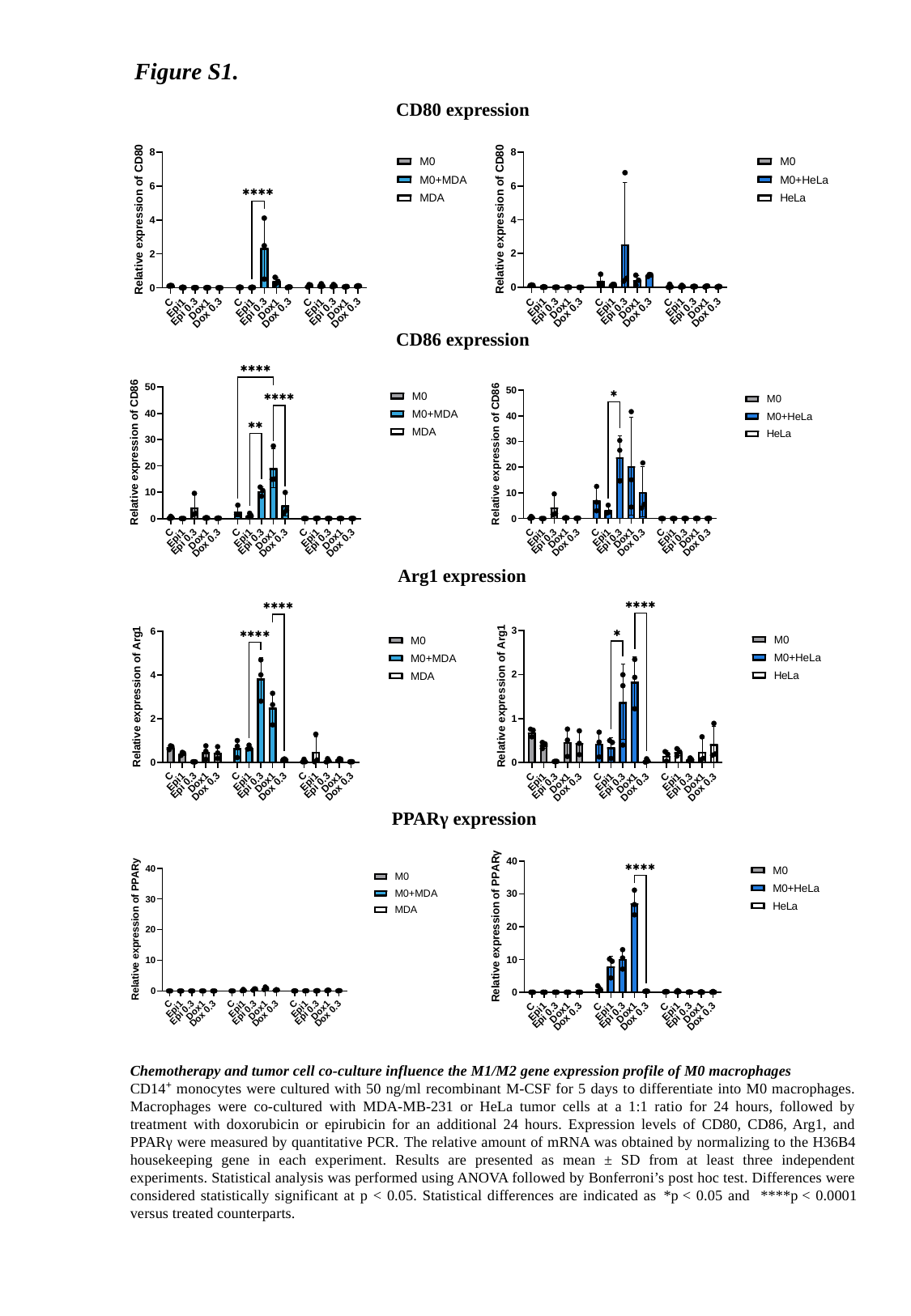

Figure S1.
CD80 expression
CD86 expression
Arg1 expression
PPARγ expression
Chemotherapy and tumor cell co-culture influence the M1/M2 gene expression profile of M0 macrophages
CD14⁺ monocytes were cultured with 50 ng/ml recombinant M-CSF for 5 days to differentiate into M0 macrophages.
Macrophages were co-cultured with MDA-MB-231 or HeLa tumor cells at a 1:1 ratio for 24 hours, followed by treatment with doxorubicin or epirubicin for an additional 24 hours. Expression levels of CD80, CD86, Arg1, and PPARγ were measured by quantitative PCR. The relative amount of mRNA was obtained by normalizing to the H36B4 housekeeping gene in each experiment. Results are presented as mean ± SD from at least three independent experiments. Statistical analysis was performed using ANOVA followed by Bonferroni’s post hoc test. Differences were considered statistically significant at p < 0.05. Statistical differences are indicated as *p < 0.05 and ****p < 0.0001 versus treated counterparts.

## Slide 2
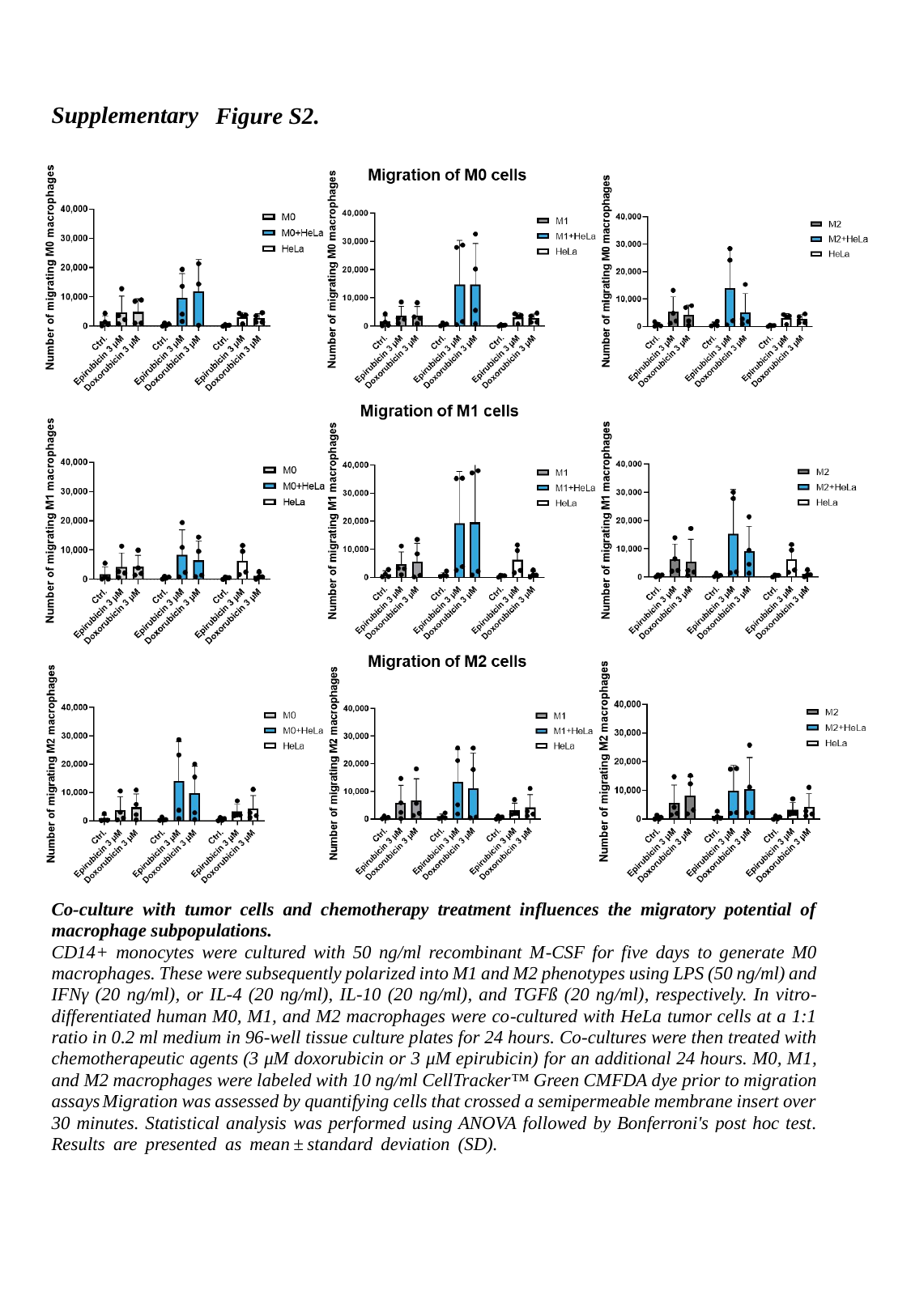

Figure S2.

## Slide 3
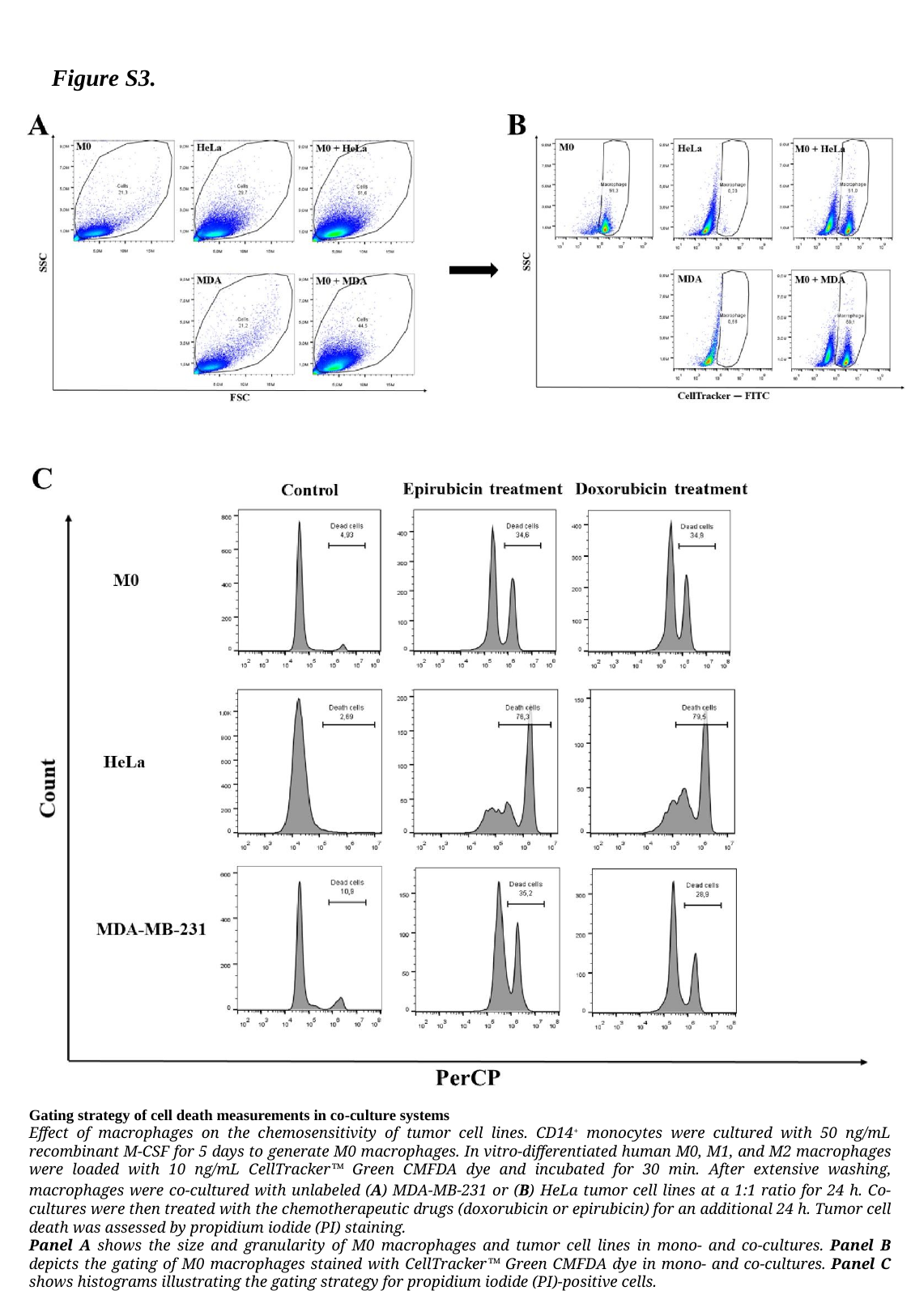

Figure S3.
Gating strategy of cell death measurements in co-culture systems
Effect of macrophages on the chemosensitivity of tumor cell lines. CD14+ monocytes were cultured with 50 ng/mL recombinant M-CSF for 5 days to generate M0 macrophages. In vitro-differentiated human M0, M1, and M2 macrophages were loaded with 10 ng/mL CellTracker™ Green CMFDA dye and incubated for 30 min. After extensive washing, macrophages were co-cultured with unlabeled (A) MDA-MB-231 or (B) HeLa tumor cell lines at a 1:1 ratio for 24 h. Co-cultures were then treated with the chemotherapeutic drugs (doxorubicin or epirubicin) for an additional 24 h. Tumor cell death was assessed by propidium iodide (PI) staining.
Panel A shows the size and granularity of M0 macrophages and tumor cell lines in mono- and co-cultures. Panel B depicts the gating of M0 macrophages stained with CellTracker™ Green CMFDA dye in mono- and co-cultures. Panel C shows histograms illustrating the gating strategy for propidium iodide (PI)-positive cells.

## Slide 4
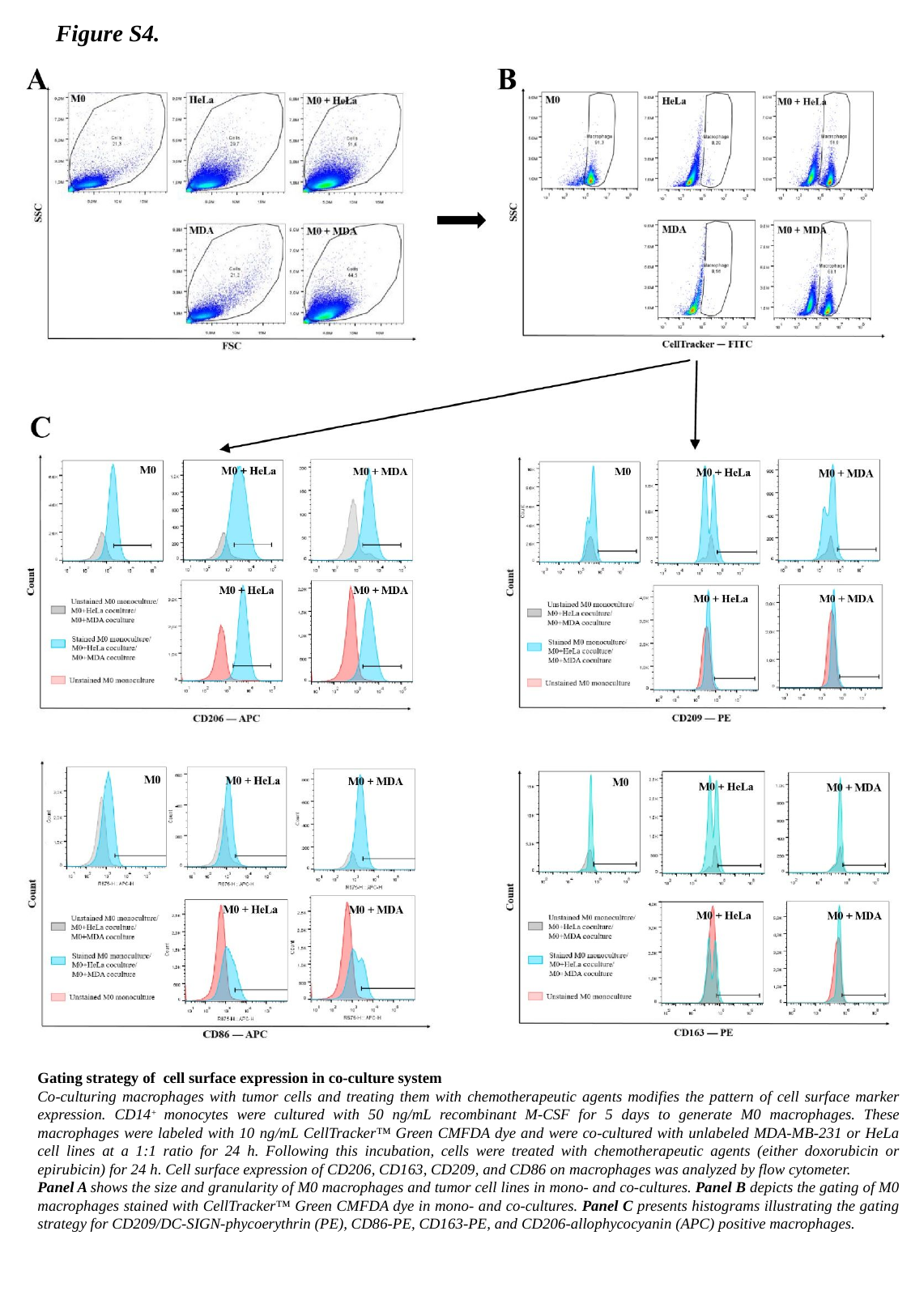

Figure S4.
Gating strategy of cell surface expression in co-culture system
Co-culturing macrophages with tumor cells and treating them with chemotherapeutic agents modifies the pattern of cell surface marker expression. CD14+ monocytes were cultured with 50 ng/mL recombinant M-CSF for 5 days to generate M0 macrophages. These macrophages were labeled with 10 ng/mL CellTracker™ Green CMFDA dye and were co-cultured with unlabeled MDA-MB-231 or HeLa cell lines at a 1:1 ratio for 24 h. Following this incubation, cells were treated with chemotherapeutic agents (either doxorubicin or epirubicin) for 24 h. Cell surface expression of CD206, CD163, CD209, and CD86 on macrophages was analyzed by flow cytometer.
Panel A shows the size and granularity of M0 macrophages and tumor cell lines in mono- and co-cultures. Panel B depicts the gating of M0 macrophages stained with CellTracker™ Green CMFDA dye in mono- and co-cultures. Panel C presents histograms illustrating the gating strategy for CD209/DC-SIGN-phycoerythrin (PE), CD86-PE, CD163-PE, and CD206-allophycocyanin (APC) positive macrophages.
